# Dependent on our dependents: advancing child health through transdisciplinary team science to sustain social programs

**DOI:** 10.1038/s41390-024-03344-8

**Published:** 2024-07-03

**Authors:** Matthew M. Davis, Mary B. Leonard, Lisa Chamberlain

**Affiliations:** 1 Nemours Children’s Health, Wilmington, DE USA; 2 Department of Pediatrics, Stanford University School of Medicine, Stanford, CA USA

As an aphorism, “Children are our future” conjures up images of today’s children all grown up, decades from now. As future adults whose wages will be taxed to fund social programs such as Social Security, Medicare, and Medicaid, today’s children will ultimately sustain social programs on which today’s working-age adults will depend for their basic needs as they reach their senior years. In this context, we see a rapidly emerging imperative for a paradigm shift in the rationale and support for a national research initiative to advance child health: to safeguard the intergenerational social contract on which an aging population progressively relies for their health and well-being.

The prospect of relying on today’s children as a future group of working-age adults to sustain large- scale social programs for an enlarging group of senior adults is increasingly dubious. The proportion of the US population over 65 is expected to exceed the proportion under 20 years old within the next decade. These trends will adversely affect the old-age dependency ratio,^1^ which is the number of individuals aged 65 and over per 100 people of working age (20–64). Many industrialized nations are experiencing similar phenomena in their rapidly aging populations (Table 1). However, the stakes in the United States are fundamentally different because, compared with other countries, the U.S. depends substantially more on individual taxpayers (predominantly working-age adults) for its tax revenue.^2^ Consequently, social programs benefiting senior citizens that rely on tax revenue are at even greater risk in the U.S. than in peer nations as the old-age dependency ratio climbs.

**Table 1:** Comparison of old-age dependency ratios for the United States and other selected Industrialized nations, in 2024 and projected in 2050.

Nation	Old-age Dependency Ratio in 2024	Old-age Dependency Ratio in 2050	% Relative Change Projected from 2024 to 2050
United States	32.2	40.4	+25.5%
Canada	34.6	44.9	+29.8%
France	39.0	54.5	+39.7%
Germany	42.4	58.1	+37.0%
India	11.9	22.5	+89.1%
Italy	41.7	74.4	+78.4%
Japan	54.9	80.7	+47.0%
People’s Republic of China	20.6	47.5	+131%
United Kingdom	34.8	47.1	+35.3%

Data source: Organization for Economic Cooperation and Development. Old-age dependency ratio.^1^

This challenge is also worsening in the United States because mortality among our youngest generation is climbing: in the most recent national data, infant mortality increased from 2021 to 2022 by the largest year-over-year increase since 2002.^3^ Countering this trend will require expertise from a wide range of fields working together to address this complex issue. Transdisciplinary team science could play a critical role in slowing or perhaps even reversing the growth of the old-age dependency ratio, by informing and advancing efforts to increase the number of children who will become working-age adults of the future.

State-by-state differences in infant mortality suggest clear opportunities to improve child survival in the U.S., by setting the goal of reducing infant mortality in all states to the level of the state with the lowest rate—currently North Dakota. However, such improvements will not be easy. After all, infant mortality rates vary more than 3-fold from North Dakota (288 per 100,000 live births) to Mississippi (947), for multifactorial reasons.^4^ Nonetheless, marked improvements in infant mortality at the state level are possible: for instance, North Dakota reduced its infant mortality rate from 596 per 100,000 in 2005 to its current level.^5^

We propose a new approach to pediatric research, designed as an all-states learning collaborative grounded in transdisciplinary team-science research that would identify, disseminate and facilitate implementation of state-level strategies associated with improvements in infant mortality. Public health professionals, in partnership with community and pediatric researchers and child- and family-focused clinicians, and including community members themselves, would work jointly to implement evidence-based interventions. Their goal would be to achieve parity across and equity within states for all populations in order to eliminate persistent gaps in infant mortality among racial and ethnic groups. Such an approach would require consideration of the most common causes of infant mortality (e.g., conditions related to prematurity; sepsis; congenital malformations; heart disease; unintentional injury) and their root causes, including social factors such as poverty and systemic racism, in order to be focused yet inclusive in taking collective action across states. This endeavor would benefit greatly from federal support for clinical care and research, using a national “moonshot” model commonly deployed to attack major diseases such as human immunodeficiency virus and COVID-19 and currently being employed to address the full array of cancer diagnoses.^6^

If all states were persistently and collectively successful in reducing infant mortality to the lowest rate among them—i.e., by reducing the national infant mortality rate to 288 per 100,000 live births—the effect on infant survival in the U.S. would be profound. Approximately 20,000 infants die each year in the U.S.,^7^ and if every state achieved the lowest current rate of infant mortality then approximately 9700 infants would instead survive to their first birthday—constituting an overall 49% reduction in infant mortality, per year (Table 2). If multiplied over a generation, holding birth rates and mortality rates among older children at current levels, the potential impact would be the addition of almost 175,000 adults of childbearing age to the US population every 18 years.

**Table 2:** Potential reduction in annual infant deaths, if all states improved to match the state with the lowest infant mortality rate.

State	Crude Infant Mortality Rate (per 100,000 population) (A)	Number of infant deaths in 2021 (B)	Number of infant deaths, if infant mortality rate was equal to lowest state (C)	Potential infant deaths averted (D = B-C)
AL	795.5	444	160	284
AK	757.9	70	27	43
AZ	563.5	429	219	210
AR	901.5	313	100	213
CA	403.0	1704	1216	488
CO	522.0	315	173	142
CT	512.7	167	94	73
DE	515.6	53	30	23
DC	649.9	57	25	32
FL	605.5	1268	602	666
GA	640.1	770	346	424
HI	455.4	72	45	27
ID	551.8	117	61	56
IL	561.8	743	380	363
IN	697.2	537	221	316
IA	415.5	148	102	46
KS	577.1	193	96	97
KY	645.0	325	145	180
LA	750.7	416	159	257
ME	550.6	62	32	30
MD	623.2	415	191	224
MA	343.5	228	191	37
MI	641.0	656	294	362
MN	491.3	309	181	128
MS	947.3	327	99	228
MO	576.2	394	197	197
MT	521.1	55	30	25
NE	567.3	133	67	66
NV	565.6	190	97	93
NH	463.7	53	33	20
NJ	372.9	355	274	81
NM	467.1	102	63	39
NY	410.9	868	607	261
NC	719.3	814	325	489
ND	287.5	28	28	0
OH	715.2	913	367	546
OK	719.5	336	134	202
OR	387.5	154	114	40
PA	563.6	719	367	352
RI	475.6	46	28	18
SC	775.2	421	156	265
SD	632.0	69	31	38
TN	658.7	505	220	285
TX	550.9	1985	1036	949
UT	485.0	218	129	89
VT	*	16	*	*
VA	612.1	566	266	300
WA	444.1	360	233	127
WV	707.9	121	49	72
WI	548.5	329	172	157
WY	519.8	32	18	14
**US**	**558.8**	**19,920**	**10,233**	**9671**

All infant mortality data drawn from CDC WONDER.^4^

All mortality counts and projections are rounded to nearest whole number.

The state of ND had the lowest infant mortality rate in 2021 and was used to estimate the potential reduction in infant mortality for other states if they reduced their infant mortality rates to that same level.

*Vermont infant mortality rate is specified as “unreliable” in CDC WONDER due to small numbers in 2021. Therefore, the number of infant deaths in Vermont is included in the national total in Column B but no estimates were calculated for Column C or Column D.

The bold values are the national mean (column A) and the national totals (columns B, C, and D).

Innovative pediatric research is also required to address escalating child and adolescent mortality rates. Recently, Woolf et al called attention to the increasing all-cause mortality rates for 1- to 19-year-olds in the U.S. and concluded that the causes were attributable to “manmade pathogens… of bullets, drugs and automobiles.”^8^ Making matters worse, the economic drivers of suicide, drug use and alcoholism as described by Case and Deaton in their book “Deaths of Despair”^9^ highlight the profound impact of the opioid epidemic, which is currently harming 20- to 64-year-olds in ways that also exacerbate the old-age dependency ratio. Health researchers will have a critical role to play in conducting transdisciplinary research to address the root causes of mortality for children and adolescents, in partnership with public health officials, epidemiologists, social scientists and policymakers.

Finally, all would concur that children must not only survive to adulthood but thrive in adulthood. Looking ahead, an increasing proportion of individuals 20–64 years old may not be healthy enough to work and thereby support social systems for their older community members. For example, in 2018, 77% of young people in the U.S. would not have been able to join the military if they wanted to, because they were either overweight or obese, had educational deficits, or had a criminal or drug abuse record.^10^ These same concerns may limit individuals’ ability to obtain and maintain gainful employment. Leveraging transdisciplinary research to expand understanding of how health during childhood and adolescence sets the stage for thriving in adulthood will inform evidence-based, scalable solutions in the first few decades of life for individuals, communities and populations.

In summary, saving children’s lives and ensuring they thrive as adults is a worthy priority, in and of itself. Recognizing that the need for our children to reach adulthood and thrive is fundamental to the future success of the nation’s social welfare and healthcare programs may serve as an additional, increasingly meaningful rationale. The sooner the nation recognizes the intergenerational imperative of the old-age dependency ratio, the sooner children may benefit from the consequent policy and programmatic attention that will benefit the U.S. population as a whole. Pediatric researchers have an essential role to play in supporting children’s survival and thriving into adulthood, including embracing new models of innovative transdisciplinary investigation to meet our current complex realities.
